# Suffragettes and the Stomach Tube

**Published:** 1909-10-02

**Authors:** 


### SUFFRAGETTES AND THE STOMACH TUBE.

We have no doubt that the action of the prison authorities in forcibly feeding certain refractory women prisoners during the past week—an action which has evoked animated questioning in Parliament and very unwise declamation out of it—will win many friends for the cause which these victims of "official brutality" allege to represent. To us, who stand in a measure outside the pale of suffragette or anti-suffragette criticism—since we have never consciously expressed any opinion on the merits of the movement as a whole—the present aspect of the case seems very simple. Mr. Mansell Moullin, in a letter to the daily press, has pointed out very clearly that the "hospital methods" which have been used in the case of these refractory prisoners are not hospital methods at all in the strict sense of the word.

Forced feeding by means of a stomach or nasal tube is frequently used in asylums, but there can be few occasions when the necessity for it arises in a general hospital. It is true that nasal feeding is frequently used in general hospitals, but in such cases the patients usually give the nurse every help possible, except where paralysis, or, with children at the first attempt, fright prevents them from assisting in the operation to the best of their ability. Such feeding is not forced, is certainly not brutal, and is not unpleasant, as the indignant protesters in Parliament may easily find if they have the courage to pass a tube on themselves. What makes the asylum system of forced feeding unpleasant is the fact that a fairly large œsophageal tube is used which is passed on a patient, secured in the usual manner by blankets or manual restraint—certainly never by chains—who obstinately refuses his food. Such a patient is, in many instances, incapable of comprehending what is being done on his behalf, and fights savagely, not, as is popularly supposed, because he has the strength of ten, but because he is possessed of the reckless disregard of consequences to himself and others which is so characteristic of the insane. The operation is, therefore, as unpleasant to the operators as it is to the patient, and in a sense it is "brutal," just as much as cauterising a nævus in a small child is "brutal." But in both cases there is justification for whatever unpleasantness may be inflicted. The women who have been subjected to "forced feeding" are experiencing nothing worse than has been experienced in past times by refractory male prisoners who stubbornly refused their food. The alternative is to allow such prisoners to starve, for no sensible person can ever admit that they should be released merely because they please to cavil at the prison fare supplied to them.

Hitherto no protest has been made on behalf of the male prisoners who have been forcibly fed, and it is illogical, to say the least, to demand that women, who claim to stand on the same level with men politically, socially and communally, should be accorded privileges which have so far been theirs just because of those differences, politically, communally, and socially, between them and men which they desire to do away with. On these claims and these desires we express at present no opinion, but we have no hesitation in saying that in our opinion the action of the prison authorities, provided it has been carried on with the necessary decency and care that forced feeding, especially in the case of women patients' demands, is fully justified in the circumstances. Certainly medical men know too well the mischief to health that may arise from the absurd practices often indulged in by prisoners to attempt evasions of their punishment to waste much sympathy on the foolish women at Winson Green.

